# “Sign Me Up”: a qualitative study of video observed therapy (VOT) for patients receiving expedited methadone take-homes during the COVID-19 pandemic

**DOI:** 10.1186/s13722-023-00372-3

**Published:** 2023-03-29

**Authors:** James B. Darnton, Elenore P. Bhatraju, Kristin Beima-Sofie, Alyssa Michaels, Kevin A. Hallgren, Sean Soth, Paul Grekin, Steve Woolworth, Judith I. Tsui

**Affiliations:** 1 Division of General Internal Medicine, University of Washington, 325 9th Ave, 359780, Seattle, WA 98195 USA; 2 Evergreen Treatment Services, Seattle, WA 98134 USA; 3 Department of Global Health, University of Washington, Seattle, WA 98195 USA; 4 Division of HIV, ID, and Global Medicine, Department of Medicine, University of California, San Francisco, San Francisco, CA 94110 USA; 5 Department of Psychiatry and Behavioral Sciences, University of Washington School of Medicine, Seattle, WA 98195 USA

**Keywords:** Opioid use disorder, Opioid treatment program, People who use drugs, Video observed therapy

## Abstract

**Background:**

Federal and state regulations require frequent direct observation of methadone ingestion at an Opioid Treatment Program (OTP)—a requirement that creates barriers to patient access. Video observed therapy (VOT) may help to address public health and safety concerns of providing take-home medications while simultaneously reducing barriers to treatment access and long-term retention. Evaluating user experiences with VOT is important for understanding the acceptability of this strategy.

**Methods:**

We conducted a qualitative evaluation of a clinical pilot program of VOT via smartphone that was rapidly implemented between April and August 2020 during the COVID-19 pandemic within three opioid treatment programs. In the program, selected patients submitted video recordings of themselves ingesting methadone take-home doses, which were asynchronously reviewed by their counselor. We recruited participating patients and counselors for semi-structured, individual interviews to explore their VOT experiences after program completion. Interviews were audio recorded and transcribed. Transcripts were analyzed using thematic analysis to identify key factors influencing acceptability and the effect of VOT on the treatment experience.

**Results:**

We interviewed 12 of the 60 patients who participated in the clinical pilot and 3 of the 5 counselors. Overall, patients were enthusiastic about VOT, noting multiple benefits over traditional treatment experiences, including avoiding frequent travel to the clinic. Some noted how this allowed them to better meet recovery goals by avoiding a potentially triggering environment. Most appreciated having increased time to devote to other life priorities, including maintaining consistent employment. Participants described how VOT increased their autonomy, allowed them to keep treatment private, and normalized treatment to align with other medications that do not require in-person dosing. Participants did not describe major usability issues or privacy concerns with submitting videos. Some participants reported feeling disconnected from counselors while others felt more connected. Counselors felt some discomfort in their new role confirming medication ingestion but saw VOT as a useful tool for select patients.

**Conclusions:**

VOT may be an acceptable tool to achieve equipoise between lowering barriers to treatment with methadone and protecting the health and safety of patients and their communities.

## Introduction

Opioid use disorder (OUD) is a major cause of morbidity and mortality in the United States and methadone remains a cornerstone of effective treatment. As of December 2020, there were over 311,531 patients with OUD receiving treatment with methadone at one of over 1600 Opioid Treatment Programs (OTPs) in the country, the only type of outpatient facility where this treatment can be provided in the United States [1]. Federal regulations tightly control the process of methadone treatment and require daily in-person attendance for observation of medication ingestion for at least the first 90 days of treatment.

The COVID-19 pandemic substantially disrupted routine OTP practices [2]. Frequent travel requirements for patients and often crowded waiting rooms upon arrival presented particular infection control challenges at many OTPs at the outset of the COVID-19 pandemic [3]. Recognizing both individual and public health risks, the Substance Abuse and Mental Health Services Administration (SAMHSA), which oversees federal OTP regulations, issued temporary guidelines allowing states to request blanket exceptions to allow for the provision of an increased number of medication doses for unsupervised self-administration away from OTP clinics during the public health emergency [4]. These exceptions arguably represented the most significant change in regulations around the provision of methadone in nearly 50 years.

Many OTPs rapidly pivoted their practices to allow for provision of an increased number of unsupervised “take-home” doses of medication, though uptake was variable and survey data suggests that only a minority of patients received a 14 day or greater supply of medication for unsupervised use [2, 5, 6]. Surveys of OTP providers show that while some clinicians supported the increased flexibility around dosing, others expressed concern about increased risks of medication diversion and overdose and described challenges in judging stability among patients [7–10]. Novel strategies may be needed to help navigate this new landscape of competing risks and mitigate the safety and diversion concerns of additional take-home doses.

Use of smartphone technology to allow remote, asynchronous observation of a patient’s medication ingestion has the potential to allay certain concerns around safety and diversion and provide additional structure and supervision, without the barrier of frequent attendance at an OTP. Video observed therapy (VOT) has been demonstrated to be effective in supporting adherence for people undergoing treatment for tuberculosis, another setting where directly observing medication ingestion is the standard of care [11–13]. Prior research with VOT of buprenorphine for OUD in office-based treatment programs suggests that, while VOT may not improve retention and illicit opioid use compared to treatment as usual in this treatment setting [14], it was feasible to implement [15] and acceptable to patients and providers [16].

In the summer of 2020 after the start of the COVID pandemic, a clinical pilot of VOT was undertaken among patients in three OTPs in Western Washington, operated by a single organization. This qualitative study was undertaken to explore the experiences of patients and providers who participated in the pilot to better understand perspectives on the program, key factors influencing decisions, and recommendations for program improvement.

## Methods

### Study design and population

We conducted a qualitative evaluation of a clinical pilot program of VOT conducted within three OTPs in Washington State using individual, semi-structured interviews of patients and counselors who participated in the pilot. Details of the pilot program have been previously described [17]. Between April and August of 2020, 60 adult patients already receiving methadone were offered take-home dosing with VOT using a smartphone application created by emocha Mobile Health^®^ and were instructed to record and asynchronously transmit videos of the ingestion of all methadone take-home doses. Their counselors agreed to be a part of the clinical pilot and were trained on how to orient patients to the VOT application, review videos submitted by patients, and respond to patient messages.

For this qualitative study, individuals were eligible to participate if they were 18 years or older, had been enrolled as patients at Evergreen Treatment Services during the implementation of the emergency response pilot, and had participated in the pilot defined as being provided the application for use. The five counselors who registered patients to use the application and managed/reviewed submitted medication adherence videos via the provider portal also were eligible to participate.

### Data collection

Patients and counselors were recruited and enrolled between 5/2021 and 8/2021. Flyers with an accompanying letter soliciting participation were provided to patient pilot participants at their respective clinics through the clinic’s “mail hold” system. Flyers were resent to participants who did not respond every two weeks for a total of four times. To capture a range of experiences, clinical pilot participants were split into three equal cohorts of 20 patients each based on the number of videos uploaded, and equal emphasis was placed on purposively recruiting from each VOT use tertile. Recruited counselors were also encouraged to refer participants from the pilot to take part in this study. Counselors who participated were recruited by email and phone calls. A $50 incentive was offered to patients and counselors who participated in the study. Those who agreed to participate completed individual, semi-structured, in-depth interviews (IDIs) and a usability testing session with the digital application (usability results to be described in a separate study). Interviews were conducted using a discussion guide with open-ended questions to explore: (1) experiences with in-person methadone treatment, (2) experiences with VOT including technical, logistical, and personal barriers and facilitators, (3) comparison of in-person and VOT methadone dosing including effects on communication, treatment, and health outcomes, and (4) recommendations for improving the program in the future. The interview guide was developed collaboratively by the study team through group discussions (see Appendix A for interview questions). Questions were designed pragmatically to assess perceptions of VOT that might impact future implementation and to address aspects of the patient experience of the OTP treatment model explored in prior studies in the literature*.* Given limited numbers of eligible participants, IDI guides were not piloted prior to use in the study but were continually evaluated for phrasing and content improvement during the data collection phase.

IDIs were conducted with one trained research assistant (AM) who had no previous interactions with study participants. Basic demographic data was collected at the start of each IDI. IDIs lasted approximately 30–45 min, were recorded using a digital audio recorder and professionally transcribed verbatim. Structured debrief reports that captured key concepts discussed in each IDI were completed within 24–48 h of interview completion and reviewed by the study team in real time to ensure data quality and identify preliminary themes based on prevalence and significance. We aimed to recruit 15 patients, including 5 from each of 3 subgroups defined by VOT adherence during the clinical pilot (i.e., low, medium, and high adherence, defined using tertiles of video uploads during the clinical pilot) and all counselors that were still employed at the OTP during the recruitment period. Participants were recruited until attempts to contact and invite each patient and counselor who had been involved in the clinical pilot to participate in this study had been exhausted.

### Data analysis

Interview transcripts were analyzed using a thematic analysis approach [18] to explore patient and provider experiences with the VOT program, including key influences on acceptability and how it affected their treatment experiences and outcomes. All transcripts were coded using a final version of the codebook, developed through a combination of inductive and deductive approaches [18–20]. First, an initial set of code categories was designed around usability and acceptability concepts included in the interview guide. Next, an open coding approach and memoing was used to review each transcript and identify specific influences on VOT experiences in more detail. Concepts identified by team members during open coding and transcript memos were compared between team members and used to develop a final version of the codebook. The final codebook was used in a consensus coding process, where four members of the research team (KBS, JD, EB and AM) coded the same transcripts and evaluated consistency in code application and text segmentation until agreement was reached. Subsequently, all transcripts were divided between coders and each transcript was independently coded by one member of the team. Coded transcripts were then reviewed by another member of the coding team, discrepancies noted, and resolved through group discussion. Queries and code co-occurrence tables were used to extract key influences on participant experiences with the VOT program and recommendations for improving future implementation of VOT. The coding team included two addiction medicine clinicians (JD and EB), a social scientist experienced in working with people who use drugs (KBS), and a trained research assistant with a health background who also conducted the interviews (AM). JB and EB work directly with patients with opioid use disorder, and interface frequently with the OTP treatment system, including the clinics where the study was conducted. None of the coding team had prior relationships with interview study participants. Dedoose (version 9.0) was used to support qualitative data management and analysis.

## Results

Interviews were conducted among 12 of the 60 patients who participated in the clinical pilot and 3 of the 5 counselors. Characteristics of the patients who participated in the IDIs, as well as of the overall participants in the clinical pilot, are shown in Table 1. IDI and patient populations were fairly comparable in relation to age, gender, race and ethnicity. IDI participants were between 30 and 64 years of age, primarily identified as male (75%) and white (75%). Patient IDI participants included two with low video use, 6 with medium use, and 4 with high use.

**Table 1:** Description of study patients who participated in the study

		Interview participants (n = 12)		Pilot participants (n = 60)	
		N	%	N	%
Age	< 30	0	0	6	10
	30–49	5	42	34	57
	50–64	7	58	20	33
	65 +	0	0	0	0
Sex	Male	9	75	29	48
	Female	3	25	31	52
Race*	American Indian or Alaskan Native	2	17	5	8
	Asian or Asian American	0	0	0	0
	Black or African American	0	0	1	2
	Native Hawaiian or Pacific Islander	1	8	0	0
	White	9	75	51	85
	Unknown or another race	0	0	3	5
Ethnicity	Hispanic or Latino	0	0	3	5
	Not Hispanic or Latino	12	100	48	80
	Unknown	0	0	9	15
Homeless**		0	0	14	23

^*^Non-exclusive category

^**^Two interview participants indicated living with family

Demographics for counselors is not reported to preserve confidentiality, given the small sample size (n = 3)

**Fig. 1:**
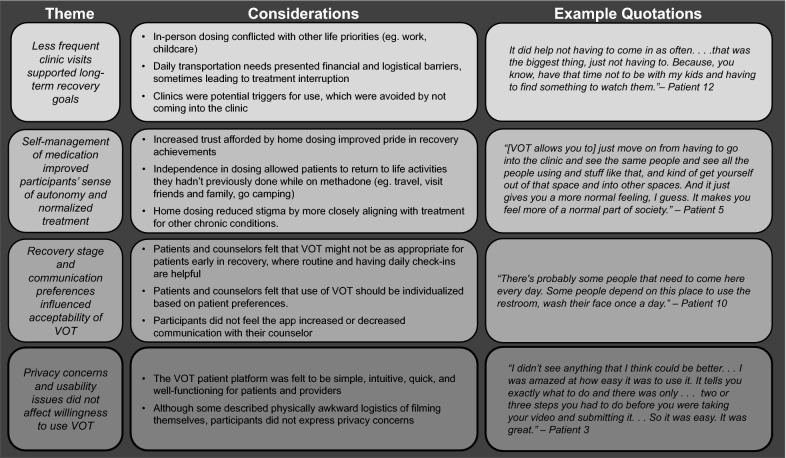
Map of themes and exemplar quotations

Overall, participants were enthusiastic about VOT, as an alternative to traditional in-person dosing options. Our analysis team identified four major themes (Fig. 1) to explain participant experiences with VOT that may inform how VOT is used in future programming.

### Theme #1: Less frequent clinic visits supported long-term recovery goals

All patients interviewed expressed that the intensity of daily in-person dosing placed a significant burden on them that limited their ability to be successful with treatment. When compared to in-person dosing, video-observed dosing was almost universally celebrated by the study participants as a significant convenience that overcame many of the challenges associated with daily in-person dosing.

*“It almost feels like a punishment coming in every day. There's not even a probation that’s that intense, you know? It’s just ridiculously intense. And almost unsustainable without assistance from somebody. For me, it’s damn near impossible to come here every day. I managed to. But if I could’ve done [VOT] from the beginning, it would've made methadone treatment long-term a lot easier on me.” – Patient 10.*

For in-person dosing, many described having to structure their days around their visit to the clinic, which came with substantial opportunity costs and interfered with other life priorities. When participants had to balance other life priorities with in-person treatment, several participants described treatment interruptions or discharge from treatment when they chose to prioritize other responsibilities. Participants particularly described how daily in-person dosing interfered with employment, childcare responsibilities, attending to other medical needs, traveling, and visiting friends and family. Related to employment, in-person dosing led to missing work, being late to work, and missing opportunities for temporary work, leading to challenges to maintaining employment, a key aspect of successful recovery for many patients.

*“[In-person daily dosing] really restricted what I [could get through] the temp service as far as jobs. Because they open at 5:30. So if you're not there at 5:30, those first 30 minutes is when they hand out all the jobs. So I’d have to come in for scraps after that . . . But yeah, when I got carries, it really made this place a lot more bearable.” – Patient 9*

For many participants, daily transportation to the clinic was a major barrier. This was especially true for those with mobility issues or chronic pain that made the daily commute more burdensome. For some, the costs associated with transportation were prohibitive, while for others, the inconsistency of public transportation created constant daily anxiety and a feeling of always needing to “*fight the clock*.” The logistics and emotional stress associated with daily transportation to clinic were relieved by the opportunity to use VOT.

*“At the time, I was still living close, but because of the pandemic I would have to ride the city bus to go [to the clinic]. And then, the bus schedules, they weren't running as much. And they weren't taking full passengers. . .There were several times that I would go to dose and I would have to wait till like the third bus. So I would be sitting there waiting with a bunch of people that I really didn't want to be waiting with. And in a situation I didn't really want to be. So the app just really made it a lot better for me. . .it just made it easier for me to stay sober.” – Patient 6*

Of note, only one patient, who self-identified as immunocompromised cited avoiding possible exposure to infectious diseases (e.g., COVID-19) by less frequent clinic visits as a benefit of video observed dosing.

For some participants, less frequent travel to clinic aided their recovery because it allowed them to purposefully avoid triggers to relapse that they might encounter at the clinic. One patient referenced the area around the clinic as *“an open drug stock exchange*,” while another described having to *“dodge”* the *“methadone mile”* when visiting clinic. For some participants, it wasn’t just about avoiding the triggers, but having to continually revisit their past, that made continued visits undesirable, especially for those who were trying to, *“just to get away from [drug use] and actually let the methadone work.”*

*“It’s the location itself [which] has become . . . kind of a hub for – well, there's people trying their best for recovery and there's people that aren’t. And there's people that are okay in their opiate side, but they're smoking crack or meth. There's always a ton of pitfalls. In the beginning, it didn't really bother me. But once I got clean, it started to bother me just being around a lot of people that weren't clean.” – Patient 1.*

Finally, several participants described feeling unwelcome and judged during clinic visits. They described feeling like they were always under suspicion of doing something wrong, and noted how the stringent rules at the clinic made them *“feel like they’re being treated kind of like children.”* For these patients, minimizing in-person dosing days allowed them to avoid stigmatization and judgement from clinic staff or conflicts with other patients.

*“There are so many rules . . .For example, you cannot pass a piece of paper with a phone number on it to someone, even if they know it's a piece of paper with a phone number on it. If you pass anything, it's considered drugs. . . . Or, if someone passes a cigarette, even if they know it's a cigarette, it's still an automatic write-up, and that kind of thing..” – Patient 9.*

### Theme #2: Self-management of medication improved participants’ sense of autonomy and normalized treatment

For many participants, self-administering medication increased flexibility in how to structure their day. One participant described how VOT allowed him to take the medication on a full stomach to avoid the nausea and vomiting he often experienced when dosing in-person, since he no longer had to rush out of the house before eating. Another participant described how VOT dosing allowed him to start his day earlier, by providing him with the slow, relaxed time he needed each morning before methadone kicked in.

*“My body isn’t really up and going until 20 minutes after I dose. I can drink coffee, but I’ll still be yawning. I’ll be in my withdrawal. But the best time for my house to the clinic is half hour to an hour… the days that I was able to just roll over and dose, it was great. I’d start my day right then and there….It was the very first bricks of having a structure. Because my life was very chaotic before.” –Patient 1.*

Some participants also described how increased take-homes with VOT improved their self-worth and agency. VOT allowed them to be *“in a position where I can start gaining some trust back,”* feeling like it is important to *have “people feeling like they can be accountable, in some ways, on their own.”*

Several participants described how storing and managing their own medications helped treatment feel more “normal:”

*“I haven’t used drugs in years. So [participating in the pilot] really didn’t help in that way . . . I didn’t go back to using or anything. And didn’t want to. It just made it easier . . . The closer you can come to having a normal life, the better off you are, you know? It made it a little closer, you know?” – Patient 11*

Others expanded on the notion that the self-management of medications also made them feel more “normal” by allowing them to participate in activities they had not been able to while on methadone—traveling “*four states away*,” visiting friends and family, and going camping. Some participants expressed how this normalization of the treatment experience might affect their long-term plans for treatment:

*“. . . before, I was stressing, like okay, I only need to be on methadone X amount of years. I'm going to get straight and I'm going to wean off, yadda, yadda because I don’t want to be down there every day and all these other things. But now I'm like, okay, now it’s a normal medication. Now I can take my medication in the morning and go about my day, just like with my antidepressants.” – Patient 1.*

Participants did not indicate that use of VOT affected how likely they were to take their medication compared with in-person dosing. They overwhelmingly reported that they took medication only as instructed. One patient indicated that VOT helped him maintain regular adherence and transition to greater take-home responsibilities that were fast-tracked because of the pandemic:

*“It made the transition a little bit better I think, instead of just having all my doses and nothing to do, not knowing—you know? So [VOT] kind of helped me just kind of remember, keep it in the front of my mind.” – Patient 4*

### Theme #3: Stage of recovery and communication preferences influenced acceptability of VOT

While the large majority of patients expressed enthusiasm for VOT as an alternative to in-person observed dosing, there was some acknowledgement that it may not be a helpful option for other patients, particularly those early on in their recovery. One participant indicated that *“an earlier version”* of himself might have *“taken advantage of”* VOT by feigning ingestion. Another described how the structure imposed by in-person daily dosing early in recovery is helpful by creating a routine and providing supportive interactions with others, and noted that this critical support would be absent if all patients started with VOT.

*“When I first started, I was very depressed, and so going somewhere, making myself go somewhere every day, and having someone to see and interact with, helped me a ton. And I believe that has just done so much for me.”*

—Patient 9.

Similarly, a counselor also reflected on her perception that in-person dosing provides a helpful scaffold for daily structure for patients with chaotic lives early in treatment.

“*[T]hat's part of the thing about them coming into the clinic every day is, you know, some people actually have a ritual of just coming in the clinic every day. Their life’s a train wreck outside of here, but for whatever reason, they come here, and they dose every day. Part of it is because it's methadone, and because they're getting the meds that they want, and they're not having to detox. And the other part of it is just the ritual of actually coming in and connecting and doing that.”*

—Counselor 1

For other participants, not necessarily in early recovery, physically coming into the program was an essential part of their recovery routine. One participant who appreciated in-person interactions noted how getting two weeks of take-home medication at a time may be less helpful for him.

*“I still wanted to keep this place fresh in my mind because it’s still part of the program with counselors and stuff. I’d forget about appointments if I only came in every other week.”*

—Patient 10.

Participants described varied expectations and experiences communicating with counselors as a part of their recovery process. For some, communication with counselors was a key aspect of recovery, while others valued communication less. Overall, participants did not feel like the app changed communication substantially or affected their relationship with their counselor. A few participants did describe an aspect of personal connection through the app, which they felt helped them relate more to their counselors and provided an additional level of motivation early on in recovery.

*“I think, if anything, it kind of felt like I was giving [my counselor] a peek into my home life and stuff. Kind of, more personal, more open. I wasn't super early in my sobriety, but it did also feel like, I don't want to say it helped keep me clean or anything. But it was definitely, like, oh my counselor’s going to see this. I definitely don't want anything going on in the background that she wouldn't like….. I think it just kind of made me, maybe, be more open. And it definitely made it feel more personal.” – Patient 5.*

### Theme #4: Privacy concerns and usability issues did not affect willingness to use VOT

In general, the VOT patient platform was felt to be simple, intuitive, quick, and well-functioning. Some participants reported instances where video uploads didn’t occur or were delayed which they attributed to internet connectivity issues.

*“I liked the fact that I could be anywhere and show myself taking my dose….If I didn't have Wi-Fi, it might be hard. But, you know, I can't think of any place, any situation where it would be hard to get to a spot with Wi-Fi, even if I didn’t have it.” – Patient 9.*

Some described physically awkward logistics of filming themselves and making sure the video satisfied requirements (e.g. medication bottle could be seen). While some participants reported feeling awkward or “camera shy”, participants generally did not express privacy concerns. When questioned, some participants indicated that they felt more comfortable knowing that their counselors were reviewing their videos as opposed to “*just a random person that works for a random company.”*

*“I felt pretty comfortable, especially knowing that it was going mainly to my counselor and then, you know, the people in the app had access to it if there was ever a problem or something. But it wasn't, you know, just whoever could see it. So I did feel, I felt comfortable using it. I never felt like my information was in jeopardy or anything that.” – Patient 5*

For others, they noted that they didn’t have a preference on whether it was their counselor or someone else reviewing their video submissions, “*as long as they’re doing it properly*.” At the time of the interview, a few participants had not realized that it was in fact their counselors reviewing their video submissions.

Participants also had the ability to text their counselors through the app. Their use of the in-app text function generally had to do with logistics of using the app, such as advice on taking and uploading videos. Sometimes app communication was used for appointment reminders while other times text communication allowed the patients to know that their videos were being reviewed by their counselor, which they found reassuring.

There was a small minority for whom using the technology itself created unwelcome anxiety. A few participants expressed concerns that the technology wouldn’t function correctly and wondered what effect a missed submission would have on their treatment plan. For one participant, this concern was salient enough that he would not want to participate in VOT in the future.

*“I don’t want to have to worry about my phone not working or breaking. I have bad luck with the screens on my phone, breaking them because I work. If the screen breaks, the phone doesn’t work. And I've replaced this phone four times last year. [T]hat means there's going to be one morning every time where I wouldn’t have been able to use the app. And four times in a year, that might’ve been enough for them to say I wasn’t [able to continue with home dosing] – that would just be an unnecessary stressor.” – Patient 10.*

The counselors also expressed that the provider interface was simple and intuitive. They indicated that initially setting a patient up for VOT could be time intensive, but daily video review and text communication was not. One counselor expressed her preference that the provider portal be incorporated into the clinic’s EHR for documentation purposes. While providers did not express usability concerns, one did express initial discomfort that observing medication adherence, which was not part of her usual scope of practice as a substance use disorder professional.

*It was kind of uncomfortable, I guess, that normally it's our nurses who determine at the window who can, at that point, ingest their dose safely or not. So, I felt like I had my own criteria and awareness of their dosing appropriateness. But I also felt like a little bit like inexperienced, I guess. – Counselor 2.*

## Discussion

In this qualitative study, we explored the experiences of patients and counselors at an OTP who engaged in a pilot program of a smart-phone based video-observation of methadone ingestion. Overall, patients and counselors largely perceived benefits over traditional treatment experiences, particularly with regards to cutting back on the need for frequent travel to the clinic and how this allowed patients to better meet recovery goals by avoiding potential recovery stressors and drug use cues in the OTP environment. Participants described how VOT increased their autonomy, allowed them to keep treatment private, and normalized treatment to align with other medications that do not require in-person dosing. Participants did not describe major usability challenges or privacy concerns with submitting videos, and some voiced feeling a greater connection with their counselor. Counselors expressed some frustrations with initial logistical challenges using the provider portal; however, in general saw VOT as a useful tool for select patients.

This study provides additional support for the emerging role of technology in providing a broader range of models for treatment that provide greater flexibility and patient-centeredness while retaining features that provide accountability and ensure patient safety. Prior research on VOT for buprenorphine has demonstrated feasibility and acceptability [15, 16]; however, demand may be limited in buprenorphine treatment settings since directly observed therapy is not a federal requirement as is the case for methadone. A recent study describes a model of care using VOT coupled with a device allowing for timed dispensing of methadone [21]. Such technology provides a greater level of safety and diversion control, yet may be more expensive and difficult to scale. Regulatory changes allowing for more flexibility in the provision of unobserved doses of medication in the wake of the COVID-19 pandemic have led to unprecedented changes in how care is delivered to patients at OTPs across the country. While observational studies suggest no increase in methadone-related poisonings for OTP patients or at the population level [6, 22–24], some patients and providers may prefer to retain the component of observed therapy without incurring the inconvenience and exposure risk (both to transmissible infections and to substance use) of coming to the OTP in-person [7, 25–27]. VOT is a tool that can provide an additional alternative in between the extremes of in-person observed dosing and fully unobserved dosing. Given the range of patient preferences and lived experiences, more choices are needed to achieve optimal outcomes.

There is hope among patients, clinicians, and advocates that changes in the regulations of OTPs initiated during the COVID-19 pandemic will lead to ongoing changes in how care is delivered [3, 28–30]. At the same time, this hope must be balanced with concerns that an increase in access to unsupervised methadone may create medication toxicity and diversion issues. The new treatment environment has created other concerns among OTP providers – that there is a lack of consensus on criteria for defining patient stability and ability to safely manage take-homes, that patients who benefit from more structured care may be harmed by more liberal provision of unsupervised medication, and that the new-found flexibility in the provision of take-homes may exacerbate disparities in health outcomes [2, 7, 9, 31, 32]. Innovative approaches to reduce the burdens of in-person observed dosing while balancing potential concerns about adverse medication effects or diversion from unsupervised dosing will be needed for this new treatment landscape. Our study supports the emerging literature that VOT approaches are well-received and can be adopted by a subset of patients to help achieve such a balance.

This study has several limitations. As part of our evaluation we assessed the trustworthiness of the results using criteria described by Guba and Lincoln [33]. Participant responses were largely rich and descriptive and generally consistent in their overall program assessments, which supports the credibility and dependability of the findings. Selection bias and the small number of participants, however, could also negatively affect the study’s credibility and generalizability. Our original planned sample size for patients was for 15 and we had hoped to include equal representation from high, medium, and low app utilizers. However, we were only able to recruit 12 participants total, of which four, six and two were in the high, moderate and low use groups respectively. This may have missed incorporation of the full range of experiences, as participants who used the app more regularly are likely to have had a more favorable opinion of it. Future research should solicit perspectives from patients with low or inconsistent use of VOT or who decline to use VOT entirely. Similarly, two counselors were no longer actively working at the site and could not be reached for participation, limiting our counselor sample size.

Overall, our sample did not represent the full range of racial and age diversity within the methadone treatment program populations. Additional research should prioritize soliciting perspectives from younger and older adults, and members of racial and ethnic minorities. Our study participants largely expounded on the benefits of VOT as compared with in-person observed dosing, the standard treatment at the time. As unsupervised take-home dosing has become more common, further studies exploring VOT technology for medications for OUD treatment should examine outcomes and attitudes around VOT compared with non-observed dosing as well.

## Conclusion

This qualitative study suggests that VOT may be an acceptable tool to achieve equipoise between lowering barriers to treatment with methadone and protecting the health and safety of patients and their communities. Further prospective research should evaluate whether VOT and other innovative delivery devices that serve to create more flexible models of treatment may facilitate increased engagement in methadone treatment.

## Data Availability

Data reported in this paper are available upon request.

## Interview questions—patients

### Engagement questions

1. Please tell me about your overall experience receiving methadone treatment.

**PROBE**: Tell me about your experience coming into clinic to dose at the window.

2. Last spring and summer, you had the chance to use the emocha mobile app for video-confirmation of home methadone dosing. What did you think of that experience?

*If participant states they did not use the app, skip to question 10*.

**PROBE**: Tell me what you liked or did not like about using an app for video monitoring.

3. How comfortable did you feel submitting video recordings of yourself through the app?

**PROBE:** Tell me about any issues you faced while submitting a video.

**PROBE**: Were there privacy concerns?

**PROBE**: Tell me about any technical issues you may have faced.

### Exploration questions

4. How did using the app for video confirmation compare to traditional in-person confirmation of methadone dosing?

**PROBE**: Tell me about which method you prefer. Why do you prefer that method?

**PROBE**: If there were no pandemic, how would you feel about using this app?

5. How did using the app for video confirmation change your daily routine?

**PROBE**: How did it change the frequency of your visits to ETS?

**PROBE**: How did it affect your treatment experience?

**PROBE**: How did it affect your substance use (i.e. drugs and alcohol)?

6. Tell me about your experience communicating with your counselor when you were using the app.

**PROBE**: How did video-confirmation of home methadone affect your relationship with your counselor?

**PROBE**: How do you feel about who reviews your video submission?

**PROBE**: How important is it that someone you know reviews the videos?

7. How has use of video-confirmation of home methadone dosing affected your treatment plans?

**PROBE**: How has it affected your plan to continue methadone treatment?

8. How, if at all, did video confirmation of take-home methadone doses affect how you took your methadone?

**PROBE**: Did you ever take the medication in a way that was different than instructed? (eg. Doubling up, taking less, giving medication to someone else? Just as a reminder, everything you tell us will remain confidential).

**PROBE**: Did the expectation of submitting videos make it any more or less likely you would take your methadone different than instructed?

**PROBE**: How did not having the structure of coming into clinic frequently to dose impact your treatment experience? Your likelihood of using substances (i.e. drugs and alcohol)?

9. How do you feel about continuing to use video-confirmation of home methadone dosing in the future?

**PROBE**: Would you recommend this application to others in methadone treatment? Why or why not?

10. Tell me about the kind of patient you think video confirmation of home methadone dosing would be most helpful.

**PROBE**: Why would this type of person find this tool useful?

11. Tell me about scenarios where you think video confirmation of home methadone dosing would most helpful for patients and for clinics.

### Exit questions

12. Tell me about how the app could be better.

**PROBE**: What features did you use? Would you recommend any additional features?

**PROBE**: Would you change the app in any way? What changes would you recommend?

*If patient did not use the app*:

**PROBE**: Tell me about how an app could change your experience receiving treatment.

13. What else should we know about your experience using this application as part of your treatment?

## Interview questions—counselors

### Engagement questions

1. Thinking back to last spring and summer, what was your experience like using the emocha provider web portal for patients utilizing video-confirmation of home methadone dosing?

**PROBE**: What specifically did you like or not like about it?

2. One of the things you have been asked to do is a novel form of video confirmation that has not been part of your original workflow. What do you think about having this new role in monitoring medication?

**PROBE**: How did it fit, or not fit, into your existing roles?

**PROBE**: How did it affect your job satisfaction?

**PROBE**: How much time do you think it took to perform the tasks related to use of this app?

### Exploration questions

3. Tell me about your experience communicating with your patients over the app.

**PROBE**: What do you think about how video-monitoring of home methadone affected your relationship with your patient?

**PROBE**: What sort of things did patients communicate with you about related to the app?

**PROBE**: Tell me about your between-visit communication with patients.

**PROBE**: How did communicating with patients change while using the app?

**PROBE**: Did you feel you were able to provide any support through the communication feature of the app? What was that like?

**PROBE**: Were there things you learned about your patients through reviewing the videos?

4. Do you think the patients benefitted from use of at home video confirmation of methadone taking?

**PROBE**: Based on your experience, do you think it matters if ETS staff review the videos rather than someone from emocha? Why?

**PROBE**: Tell me about your perception of patient’s use of the app. (i.e., did they use it? Did they like it?)

**PROBE**: What kind of effect do you think it had on their overall engagement or success in treatment? Their likelihood of using substances?

5. How, if at all, did video monitoring of take-home methadone doses affect how patients took their medication?

**PROBE**: Could you tell if patients ever took their methadone in a way that was different than instructed (eg. Doubling up, taking less, giving medication to someone else?)

**PROBE**: How did it affect how patients adhered to taking their methadone?

6. How do you feel about using the app for patients as a tool to prevent diversion?

**PROBE**: How could it be more effective?

7. What do you think about seeing this technology used more broadly in OTP Settings for patients?

**PROBE**: Tell me about what would need to happen to make this work?

**PROBE**: Would you recommend this application to other counselors or providers?

**PROBE**: Tell me about the kind of patient you think video directly observed therapy would be most helpful?

**PROBE**: Tell me about scenarios where you think video confirmation of home methadone dosing would most helpful for patients and staff.

### Exit questions

8. Tell me about how the web portal could be better.

**PROBE**: Would you change the web portal in any way? What changes would you recommend?

**PROBE**: What features did you use?

**PROBE**: Would you recommend any additional features?

9. What else should we know about your experience using this platform to treat patients?

## Abbreviations

OUD: Opioid use disorder
OTP: Opioid treatment program
VOT: Video observed therapy
SAMHSA: Substance Abuse and Mental Health Services Administration
